# Emotional Campaigning in Politics: Being Moved and Anger in Political Ads Motivate to Support Candidate and Party

**DOI:** 10.3389/fpsyg.2021.781851

**Published:** 2022-01-13

**Authors:** David J. Grüning, Thomas W. Schubert

**Affiliations:** ^1^Department of Psychology, Heidelberg University, Heidelberg, Germany; ^2^Department of Psychology, University of Oslo, Oslo, Norway

**Keywords:** political advertisement, being moved, anger, emotion evocation, motivation, support

## Abstract

Political advertising to recruit the support of voters is an inherent part of politics. Today, ads are distributed via television and online, including social media. This type of advertisement attempts to recruit support by presenting convincing arguments and evoking various emotions about the candidate, opponents, and policy proposals. We discuss recent arguments and evidence that a specific social emotion, namely the concept kama muta, plays a role in political advertisements. In vernacular language, kama muta is typically labeled as being moved or touched. We compare kama muta and anger theoretically and discuss how they can influence voters’ willingness to support a candidate. We then, for the first time, compare kama muta and anger empirically in the same study. Specifically, we showed American participants short political ads during the 2018 United States midterm election campaigns. All participants saw both kama muta- and anger-evoking ads from both Democratic or Republican candidates. In total, everybody watched eight ads. We assessed participants’ degree of being moved and angered by the videos and their motivation for three types of political support: ideational, financial, and personal. The emotional impact of an ad depended on its perceived source: Participants felt especially angry after watching the anger-evoking ads and especially moved by moving ads if they identified with the political party that had produced the video. Both emotions mediated were associated with increased intentions to provide support. Importantly, if one of the two emotions was evoked, its effect on political support was enhanced if participants identified with the party that had produced the ad. We discuss limitations of the method and implications of the results for future research and practice.

## Introduction

Political advertisement is an inherent part of politics today and has been so since ancient civilizations. In addition to rallies, canvassing, billboards and telephone calls, modern political advertisement heavily relies on campaign spots that are plaid on TV or on websites, including social media, hereafter political ads.

In the United States, the number of political ad airings on television, federal and gubernational, increased by 86 percent from 2014 to 2018 (Wesleyan Media Project, 2018a). In 2020, the Wesleyan Media Project recorded over 4.9 million ads having aired for federal races this year, more than double the volume of ad airings in the 2012 and 2016 (Wesleyan Media Project, 2020a,b). It is evident that political advertisement has a wide reach that goes on to grow rapidly.

## Research on Political Advertisement

In parallel to the spending on political advertisement, research on it has been growing. Besides structural topics – like ethics and policy (e.g., Banker, 1992; Shaw and Ragland, 2000; Gelb and Bush, 2011), branding (e.g., Guzmán and Sierra, 2009; French and Smith, 2010), and technological advancements (e.g., Kaid, 2002; Mylona, 2008), this research area also investigated psychological aspects of the strategies in political advertisement (e.g., Benoit, 2000; Airne and Benoit, 2005; Dardis et al., 2008), and compared them across cultures (e.g., Kaid and Holtz-Bacha, 1995; Griffin and Kagan, 1996; Tak et al., 2007; Harris and Lock, 2010).

Particular emphasis has been on affective and cognitive responses, such as resulting liking, beliefs, and intentions. Negative ads, so-called *attack-ads*, have been shown to effectively motivate viewers (e.g., Kern and Just, 1995; Bradley et al., 2007; Phillips et al., 2008). However, research (e.g., Yoon et al., 2005) also explored their boundary conditions, like, for example, viewer-involvement and source credibility. Additionally, attack-ads were shown to have potential for causing backlash (Phillips et al., 2008). Positive ads have increasingly come into focus. Recent work shows that positive ads also influence viewers’ motivation to vote for a specific candidate or party (e.g., Seibt et al., 2019). While boundary conditions exist for the motivational effect of positive ads, too (e.g., Meirick et al., 2011), these results challenges the view that negative ads are more effective (Jasperson and Fan, 2002; Stevens, 2012). Research suggests that the most effective political campaigns make use of both types of political ads (Devlin, 1995).

Beyond mere cognitive responses, political ads also try to change preferences for specific politicians, create apprehension toward others, and influence actual turnout by fostering emotions (see e.g., Brader, 2005; Fridkin and Kenney, 2012; Seibt et al., 2019). The body of research on emotions in political ads, although gradually growing, is comparably small. Academic work on identifying the emotions’ impact has been especially limited. In the current work, we focus on two specific emotions that differ in their valence, *being moved* and *anger*, and examine their role in creating political support.

## Emotions in Political Ads

Brader (2005) researched 1,425 political ads from the years of 1999 and 2000, investigating what emotions were evoked in viewers. He found that 72% of the ads were rated to focus on emotion rather than logic. Brader assumed political advertising to significantly influence the decision making of voters, that is, besides other things also their voting behavior. Other scientists agree that emotions are important and decisive for political campaigns (e.g., Marcus et al., 2000). Politicians have realized the political utility of emotions in advertisement, too. Ridout and Searles (2011) showed when and how ads used emotional appeal and that they did this very strategically. Leading candidates were more likely to use positive emotions like enthusiasm and pride as appeals to foster support, while trailing candidates focused on negatives emotions like fear and anger to mobilize their followers.

Emotional ads can either just aim at evoking a general affect in the viewer or they serve the evocation of a specific emotion to guide voters’ attitudes toward a candidate and party (Chang, 2001). Various different emotions can be targeted. They include negative emotions like *anger, contempt, disgust*, and *fear* (Fridkin and Kenney, 2012), as well as distinctly positive emotions like *awe* or what is called *being moved* in English vernacular (Seibt et al., 2019). In the following work, we focus on two emotions in particular: being moved, which we conceptualize using Kama Muta Theory (see e.g., Fiske, 2019; Fiske et al., 2019), and anger. Both are central components of the modern landscape of political advertisement. Being moved is especially evoked by somewhat longer ads that include narrative and praise a specific political candidate (see e.g., “Progress is on the ballot” from the United States presidential election of 2016; accessible via OSF^1^). Twenty one percent of Brader’s (2005) 1,425 analyzed political ads were rated to evoke compassion, which shares relevant traits with *being moved*, and *moving* content was additionally explicitly found in viral political ads of the United States presidential election in 2016 (Seibt et al., 2019).

Anger plays a key role in the so-called attack ads. Ads can elicit anger toward a state of affairs against which the politician wants to act, but often the anger is directed at a political opponent. One half of Brader’s 1,425 ads were identified as anger evoking, which makes anger the most frequently applied emotion in the array of analyzed ads. From 2014 to 2018 the number of attack ads in the United States midterm elections has gone up by 61 percent (Wesleyan Media Project, 2018b).

### Kama Muta

*Being moved –* also *feeling moved* -was already recognized as an emotion by Darwin (1890) in his outline of emotion in human and animal (1890). However, being moved has been difficult to theorize properly, in large part due to the fact the English term is used very broadly and it has no clear facial expression, but rather shares shedding of tears with sadness. Recently, kama muta theory has been formulated to carve out a specific social emotion,^2^ termed kama muta (Sanskrit for “moved by love”). Kama muta is conceptualized as a positive social-relational emotion that is felt when close (communal) relations intensify, and motivates to engage in strengthening of communal relation. It largely overlaps with what English speakers call being moved, but there are some instances that most English speakers would not call being moved (e.g., the emotion elicited by observing intense cuteness), and vice versa there are some experiences that could be labeled being moved that are not kama muta (e.g., sadness over loss) (Fiske et al., 2017a,^2019^; Seibt et al., 2017).

While some research suggests that being moved is the result of evocations of solidarity, attachment, and generosity (Tan and Frijda, 1999) or witnessing moral acts (Haidt, 2003; Algoe and Haidt, 2009), Fiske et al. (2017a,2019) have found that being moved is connected to the intensification of communal sharing. Communal sharing refers to a type of social relations where people feel a shared essence and therefore feeling unity with others (Fiske, 2004). As a result, they are motivated to share resources with these others largely without tracking reciprocity. Kama muta is proposed to be an emotion that is simultaneously biologically grounded in an evolved blueprint and culturally complemented. It thus depends on both biological and cultural evolution. The blueprint is recognizable in comparisons across cultures (Seibt et al., 2017; Schubert and Grüning, 2021). However, it is developed differently into culturally unique concept, and then evoked by different practices, is characterized by different experiences, and covers different meanings in different cultures (Fiske et al., 2017a,^2019^). The folk concept *being moved* overlaps to a large degree with kama muta, but often includes emotional states that are not part of kama muta (e.g., sadness), and does not exclude others that would be kama muta (e.g., what is felt after seeing a really cute animal). Nevertheless, using the experience label “feeling moved/touched” is a useful component of any scale that measures kama muta (Zickfeld et al., 2019).

The emotion of kama muta is comprised of five components: appraisal, label/ing, physiology (=sensation and feeling), motivation, and valence (Zickfeld et al., 2019). Evidence on three of these characteristic elements dovetails with work on feeling moved (Cova and Deonna, 2014; Menninghaus et al., 2015; Fiske et al., 2017a). First, the emotion contains positive affect. Second, it comprises specific sensations, like, for instance, goosebumps, chills, and feelings of warmth in the chest. Third, the emotion motivates prosocial and altruistic behavior.

A stimulus (e.g., a political ad) should evoke *kama muta* if the stimulus includes the intensification of communal sharing, for instance a communal relation that is in danger, and a subsequent confirmation, renewal or triumph of this endangered communal sharing (Fiske et al., 2017b). It is also plausible that perceivers will *be moved* if a problem is introduced and communal relations are emphasized to tackle this problem, therefore perceivably intensifying communal sharing relations. To make this intensification of communal sharing happen, kama muta content emphasizes certain words and phrases (e.g., “we/our,” “helping each other,” and “together”). Displayed video images facilitate the same effect; sequentially showing pictures of several different people in the same context guides the viewer to conclude a notion of “we.” Combining moving visualized actions (e.g., people helping each other), music sequences and titles (e.g., “Bridges”), videos have an optimal basis for intensifying the communal sharing relations between perceiver and the perceived person.

### Anger

According to Carver and Harmon-Jones (2009), two fundamental features constitute the emotion of anger: first, its appraisal of a situation (Smith and Ellsworth, 1985; Ortony et al., 1988; Roseman, 1991; Scherer et al., 2001), and, second, the motivational processes behind anger (Arnold, 1960; Frijda, 1986; Roseman, 1991; Carver and Scheier, 1998; Davidson, 1998; Cacioppo et al., 1999). For the former feature, there exists the basic distinction of emotions being appraised as either positive (e.g., pleasant) or negative (e.g., aversive) in a situation (Lazarus, 1991). *Anger* is categorized as a negative emotion in general (Harmon-Jones, 2004). However, there are many different emotions which are generally categorized as negative. In the past, additional emotion components besides an emotion’s appraisal were sought to distinguish anger from other negative emotions: for example, a violation of thought rules (e.g., Frijda, 1986; Mascolo et al., 2000), the experience of and focus on blameworthiness (Ortony et al., 1988) or the experience of being hurt by an intentional act of someone (Frijda, 1986). Carver and Harmon-Jones (2009) argued that all these additional aspects of an event besides valence do the same thing; they all motivate to remove (e.g., Depue and Iacono, 1989; Fox, 1991), the second fundamental feature characterizing anger. In more detail, Carver and Harmon-Jones (2009) argue that “anger often promotes the effort to remove the violation of what ‘ought’ to be.” This can lead to the motivation to change someone’s behavior (Fischer and Roseman, 2007) or, more generally, it leads to the motivation to reopen the closed path to a desired goal (Frijda, 1986). Building on this, Carver and Harmon-Jones lay out a range of evidence that anger is connected to an approach-oriented motivation rather than an avoidance-oriented one; like the majority of emotions described to have a negative valence (e.g., fear, disgust, or shame). The authors conclude that this makes anger an approach-related affect (Carver and Harmon-Jones, 2009). Additionally, Neumann (2000) showed that feelings of anger presuppose that the cause of the negatively experienced event is external. External attribution exerts feelings of anger in contrast to, for example, guilt, which is mainly related to internal attribution. This further supports the characterization of anger as approach oriented. An angry mind might, before all else, approach the problem because it is perceived as an external one.

### Emotions From Political Ads Depend on Existing Social Relation

Seibt et al. (2019) showed, with the example of kama muta, that the elicitation of emotions from a political ad depends on the viewer’s degree of prior preference for and identification with the candidate or party presented in the spot. The authors showed participants real political ads from the United States election in 2016, from Clinton and Trump, and asked them about their preference for one or the other candidate and party beforehand. Their studies showed that Clinton voters were much more moved to tears by Clinton ads than by Trump ads and vice versa. The authors subsequently showed that the elicitation of the emotion kama muta predicted how motivated a viewer was to support the shown candidate with her or his vote.

## The Current Research

The present study serves as an extended replication and validation of the results of the study of Seibt et al. (2019) in the United States presidential election in 2016. We provide evidence on the robustness of the emotional and motivational effects but in a new setting of political advertisement in the United States, namely the mid-term instead of the presidential elections which differ in multiple aspects. Beyond providing evidence on the validity of these findings, the present study contributes two new insights to the literature on motivational effects of political advertisement. First, to our knowledge, we are the first to compare the evocation and the motivational effects of a social-relational emotion, kama muta, with the prominent emotion of anger in the context of political advertisement. Second, we use political ads that are substantially shorter than ads used in previous research so far (length of 35 s on average), investigating the spectrum of effect sizes of emotional and the subsequent motivational effects of political ads.

For both emotions, being moved and anger, we hypothesized two effects: (1) the enhanced evocation of the emotion by watching selected political ads that match a person’s political preference and (2) an effect of this evocation on the participants’ motivation to support the political candidate or party that commissioned the watched ad. We pre-registered all hypotheses^3^.

### Being Moved

An intensification of an interpersonal relation should especially be facilitated for peers considered as ingroup. Whether a political spot moves the viewer depends on the match between the ad’s presented core values and the viewer’s own worldview. As Seibt et al. (2019) already theorized, it also depends on the social relation between viewer and the candidate or party presenting the ad. That is, the prior identification with and preference for a candidate or party should influence the elicitation of kama muta, too, because communal relations are transitive (Fiske, 1992).

*Hypothesis 1.* Participants should be especially moved by an ad that has the party affiliation which the viewer identifies with most.

Additionally, with the extent to which a political ad is evoking the emotion of being moved, the participant’s motivation should increase to support the shown candidate and the associated party. We reason this prediction through kama muta theory (Schubert et al., 2016; Fiske et al., 2017a; Seibt et al., 2017, 2018). The theory assumes that the intensification of communal sharing leads to the motivation to devote oneself to the communal sharing relationships which were intensified just then. Within the political context devoting oneself to this relationship might manifest in actively voting for the candidate and in supporting the candidate by ideational (e.g., acquiring other’s votes) and financial means (e.g., donation). We test this hypothesis for the components of being moved, namely feeling, sensation, appraisal, and valence, separately.

*Hypothesis 2.* Being moved by the ad should have a significant effect on the viewers’ motivation to support the candidate or party that commissioned the moving ad.

### Anger

Anger analyses were explicitly planned and pre-registered as an exploratory approach. In this regard, we formulated hypotheses for effects on anger (hypothesis 3) and of anger (hypothesis 4) in political advertisement that matched the theoretically derived hypotheses for being moved.

*Hypothesis 3.* Participants should be especially upset by an ad that has the party affiliation which the viewer identifies with most.

*Hypothesis 4.* Being upset by the ad should have a significant effect on the viewers’ motivation to support the candidate or party that commissioned the upsetting ad.

In summary, we predict that emotion evocation is increased when the videos party affiliation and the viewers’ party preference match (H1 u. H3). We further predict that with the extent to which the two emotions, kama muta and anger, are evoked the participant’s motivation increases to support the candidate and party responsible for the ad (H2 u. H4).

## Materials and Methods

The study was run from the 27th to the 30th of October in 2018, 1 week before the midterm elections in the United States, with participants from the United States. We selected eight political ads for eight different candidates to be shown. Note that we did not match participants’ geographical location to the states or districts in which the candidates ran.

### Participants

A total of 223 American participants were recruited and reimbursed via Mechanical Turk with the goal of reaching a sample size of 150 participants based on the recommendations of Schönbrodt and Perugini (2013). They were paid $3 each. We excluded participants who indicated to identify neither with Democrats nor with Republicans. As pre-registered, we also excluded participants who guessed the Democratic Party or an independent group as the ad-commissioners of only one specific ad, the “America is back”-ad (see Text Footnote 1). It was highly evident that this ad was commissioned by the Republican Party and served as an attention check. The final sample consisted of 146 participants. 76 indicated to be male (52.1%), 69 to be female (47.3%) and one preferred not to indicate a gender (0.7%). The average age was 39 years, ranging from 21 to 72. Of those who indicated a preference, 105 of the participants identified themselves more with the Democratic party (71.9%), while 41 identified themselves more with the Republican party (28.1%). Fifty-two participants reported to live in an urban neighborhood (35.6%), 70 to live in the suburbs (47.9%), and 23 to live in a rural neighborhood (15.8%, one missing value). Regarding ethnic background, 114 of the participants indicated to be White (78,1%), 16 to be Black (11%), 9 to be Asian (6.2%), 4 to be Hispanic (2,7%), and 2 participants indicated *Other* writing they were “biracial” and “White/Native American” (1.4%). One preferred not to indicate (0,7%). In the present study, 36 out of the 50 states (+D.C.) in the United States were represented.

### Design

The design varied experimentally within participants the source of the video (Democratic or Republican) and the emotion elicited by the video (kama muta or anger videos). For each combination we showed two videos, resulting in a total of eight videos. Videos were shown in random order. Participants’ initially stated party preference was a quasi-experimental between-subjects factor (Republication vs. Democratic). In sum, the design was a 2 (video source, within) × 2 (video emotion, within) × 2 (party affiliation, between) mixed model.

### Video Material

We used the 2018 United States midterm elections as source for the video material for several reasons. We chose the environment of American politics for two reasons. First, for the validity of replication, we wanted to stay in the same cultural and political realm as Seibt et al. (2019) and second, the two-party system in the United States offers an optimal starting point for researching the evocation and effects of emotions that rely on group-perceptions. The midterm elections were focused on for two reasons. First, from a practical perspective, they were the closest large election event in the United States at the time. Second, with our findings we aimed at expanding existing findings beyond the United States presidential elections and show that we find similar effects in the smaller but highly important midterm elective events in the United States, which typically have a lower turnout and mobilize more partisan voters.

We searched political ads online that according to our judgment elicited either kama muta or anger in a clear way. Note that we did not sample stimuli from a larger sample of ads randomly. Our results should thus be viewed as proof of existence of the hypothesized effects, but they cannot be generalized to the larger body of emotional ads.

In general, moving political ads are characterized by the introduction of a problem that can perceivably be tackled by working together. This intensifies people’s feeling of communal sharing between the actors in the video, themselves, and other potential viewers. For the Democratic side two political ads stood out especially: (1) Bill Nelson for Senate and (2) Carper for Senate. The video titles “Stars” and “Bridges” already set the stage for the emotion of *being moved*. Adjectives like “together” and “helping each other” were predominant in the videos. This suits *being moved* very well. Additionally, both videos showed a wide reach across viewers side. These were the reasons why we chose these two ads for the Democratic side. On the Republican side we found two slightly shorter videos which show the same emotional direction trying to evoke a feeling of communal sharing, whose intensification is considered to be the main reason for *being moved* (Seibt et al., 2017). The one Republican video “More to Do/America is back” was especially interesting because it, at the time of selection, had over 4.5 million views on YouTube. While the second Republican video “Harvey” just had over 16.000 views at the time of selection, it exhaustively fulfilled criteria for a moving video and was therefore implemented in our study as the second political ad on the Republican side.

Because there were so many different *anger*-ads, our main criterion for choosing suitable videos was the intensity of the emotion evocation without taking the viewer-counts into account. The content of these political ads was characterized by the focus on one individual of the opposing political party that was accused of a specific wrongdoing that affects the whole community, state, or even country (i.e., money reputation). We chose two attack ads for the Democratic side, one against Barbara Comstock and the other against Mike Coffman, both politicians of the Republican party; and two attack ads for the Republican side, one against Joe Donnelly and the other against Joe Radinovich, both affiliated with the Democratic party. All four anger-ads were chosen because they showed the potential for a very intense anger evocation by an explicit and concrete accusation displayed in the videos.

In sum, we chose eight different videos. Each video is accessible on the corresponding OSF page (see Text Footnote 1).

### Procedure

After the presentation of general information about the experiment, participants were asked to report their political identification (i.e., “What party do you identify with more?”) choosing between identifying more with the Republican party, the Democratic party or neither of them. Subsequently, the respondents were shown the eight political ads in a randomized order.

After each ad viewers indicated what party affiliation they believed the video to have (i.e., “What do you think – was this an ad for a Republican, Democratic, or Independent candidate?”). Further, respondents reported (1) how *moving* and (2) how *upsetting* the content had been to them. The emotion kama muta (1) was assessed by asking questions about respondents’ feeling (e.g., “The ad was touching.”), sensation (e.g., “The ad was eye-moistening.”), appraisal (e.g., “There were people in the ad who were welcoming or being welcomed.”) and positive valence (i.e., “The ad had a positive tone.”), whereas anger (2) was assessed by asking about participants’ feeling toward the ad-commissioning candidate (e.g., “The person commissioning this ad annoyed me.”), their feeling toward the featured candidate^4^ (e.g., “The person being criticized in this ad annoyed me.”), appraisal (i.e., “There were people in the ad who were criticized and rejected.”), and negative valence (i.e., “The ad had a negative tone.”). All items were presented with a 7-point Likert scale, anchored in 1 (“not at all”) and 7 (“very much”), as is suggested for measuring kama muta by Zickfeld et al. (2019). Moreover, for every video participants indicated their motivation to support the candidate that had commissioned the seen ad, regarding voting support (i.e., “If I could vote in this election, I would be inclined to vote for the candidate or party that commissioned the ad.”), financial backing (i.e., “I think it makes sense to give money to the candidate or party that commissioned the ad.”), and ideational help (i.e., “If I knew somebody who can vote in this election, I would try to argue for the candidate or party that commissioned the ad.”). Respondents indicated their (dis)agreement with the three statements on a 5-point Likert scale, ranging from 1 (“not at all true”) to 5 (“completely true”).

Finally, participants reported if they had seen any of the eight ads before and if they had had technical complications (e.g., sound or video playback). Subsequently, respondents indicated their gender, age, ethnicity, and the country, state, and neighborhood they were living in.

## Results

### Pre-registration

We pre-registered our analyses (see Text Footnote 3). We had 146 participants in the final data set. Each participant watched 8 videos. This resulted in a sample of 1,168 observations. Our sample fell short on our pre-registered goal of 150 participants by four. We considered this number still satisfactory. We, further, pre-registered to remove every video for which its source (=commissioning party) was correctly identified in less than 66% of the cases. We didn’t have to do so with any video. According to pre-registration, how we treated wrong guesses on the other videos was dependent on the wrong guesses’ frequency in the specific cases. For the kama muta videos we found that in less than 25% of the cases participants wrongly guessed which party commissioned the ad. Therefore, we excluded all cases in which it was guessed wrong which party commissioned the specific ad and conducted all kama muta analyses with the variable *video party* (i.e., the videos’ real party affiliation). For the anger videos, we found that in more than 25% of the cases participants guessed wrong which party commissioned the ad. Therefore, as pre-registered, we conducted anger analyses twice: (1) with the videos’ real party affiliations as independent variable and all wrong guessing cases removed (as in the kama muta analysis), and (2) with the party affiliation a viewer guessed to be the video’s affiliation, *party guess*. No playback problems with the videos or difficulties with sound were reported. Thus, no additional exclusions followed.

Aligning with our pre-registration, we also checked if the items for the two different components of feeling anger (six items in total) could be aggregated into one scale. The two sub facets were anger toward the candidate and party that were (1) featured in the ad (feeling toward protagonist) and toward those that (2) commissioned the ad (feeling toward commissioner). Both sub facets showed satisfactory reliabilities (α = 0.94 and α = 0.95). The correlation of these two components, however, was higher than *r* = −0.60 (i.e., it’s absolute value was <0.60). Therefore, as pre-registered, we treat them as two independent facets measuring the feeling component of anger. The kama muta items’ internal consistency was α = 0.88 for the five sensation items, α = 0.86 for the two appraisal items. We computed average scores for these emotion components. Valence was only represented by one item for being moved and anger each, appraisal of anger was only measured by one item and the present study included no sensation items for measuring anger. The measure of motivation to support a candidate had three items, which showed high internal consistency (α = 0.94), and we thus averaged them.

### Being Moved

The analyses were carried out in SPSS, focusing on two independent variables: (1) party preference of the participant (Republican vs. Democratic), and (2) the party affiliation of the ad (Republican vs. Democratic). The dependent variables we were interested in were the strength of emotion evocation (Hypothesis 1) and the motivation to support the ad-commissioning candidate (Hypothesis 2).

#### Manipulation Check

Kama muta-ads, as expected, moved viewers more than the anger-ads did; for feeling, *F*(1,1166) = 388.45, *p* < 0.001, $\eta_{\text{p}}^{2}$ = 0.25, sensation, *F*(1,1166) = 119.07, *p* < 0.001, $\eta_{\text{p}}^{2}$ = 0.09, appraisal, *F*(1,1166) = 1780.82, *p* < 0.001, $\eta_{\text{p}}^{2}$ = 0.60, and positive valence, *F*(1,1159) = 2688.13, *p* < 0.001, $\eta_{\text{p}}^{2}$ = 0.70.

##### Hypothesis 1

**FIGURE 1:**
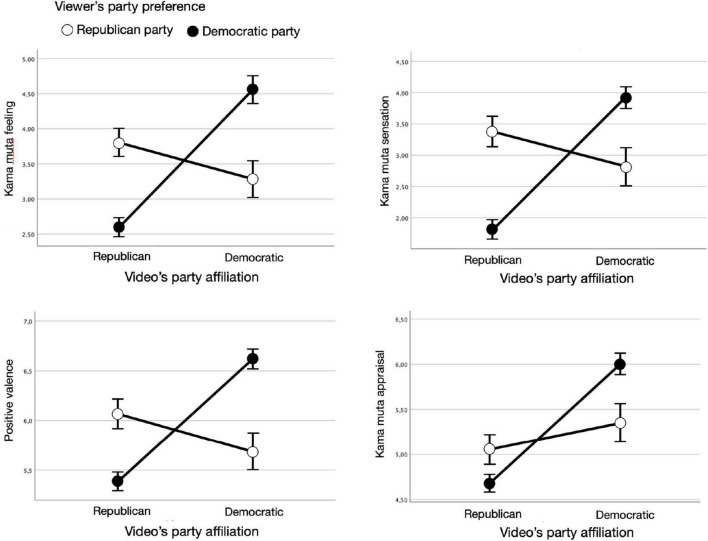
Interaction effects of video’s party affiliation and viewer’s party preference on the four components of kama muta: feeling, sensation, positive valence, and appraisal.

Subsequently, we ran four mixed models with the independent variables video’s party affiliation (Republican vs. Democratic), the participant’s party preference (Republican vs. Democratic), and video ID (1 to 4, nested within the video’s party affiliation), and the evocation of the four kama muta emotion components as the dependent variables. Note that we treated video as a fixed instead of a random factor because it has to few levels. As shown in Figure 1, the four independent analyses showed that the two factors viewers’ party preference and the ad’s party affiliation interacted significantly for all four emotion components: for feeling, *F*(1,362.0) = 48.49, *p* < 0.001, *B* = 2.14, for sensation, *F*(1,357.4) = 33.89, *p* < 0.001, *B* = −0.18, for appraisal, *F*(1,375.3) = 12.72, *p* < 0.001, *B* = 0.61, and for valence, *F*(1,364.6) = 33.62, *p* < 0.001, *B* = 1.74, respectively. Viewers felt more moved by the ad content, felt greater physical sensation, appraised the content as more communally sharing and perceived it as more positive when their party preference was the same as the video’s party affiliation (vs. not the same). The video party had a main effect on the evocation of all four components while the viewer’s party preference had no effect in all four cases.

##### Hypothesis 2

We used four mixed models with the dependent variable being the motivation to support the candidate commissioning the ad and the independent variables being the video’s party affiliation (Republican vs. Democratic), the participant’s party preference (Republican vs. Democratic), and video ID (1–4, nested). The four emotion components of kama muta (i.e., feeling, sensation, appraisal, and valence) were used as separate predictors of viewers’ motivation to support a candidate in the four models to see whether each of them predicted motivation on its own.

Before running these models, we ran one preliminary model without including the four emotion components explicitly as predictors. Here, the interaction effect of the participant’s party preference and the ad’s party affiliation on the motivation to support a candidate was significant, *F*(1,378.5) = 377.47, *p* < 0.001, *B* = 4.05. That means, if the participant’s party preference and the video’s actual party affiliation matched, the motivation to support the candidate that commissioned the ad was higher. Further, the video’s party affiliation had a main effect on the motivation to support, *F*(1,378.6) = 19.65, *p* < 0.001, *B* = −2.54. Specifically, Democratic ads were more motivating in general (*M* = 3.49) than Republican ones (*M* = 2.07). The viewer’s party preference also had a significant main effect, *F*(1,152.1) = 5.12, *p* = 0.025, *B* = −1.82.

When adding the four emotion components of kama muta as predictors (in four separate models), all four predicted motivation significantly: feeling, *F*(1,377.8) = 201.42, *p* < 0.001, *B* = 0.41; sensation, *F*(1,411.2) = 214.29, *p* < 0.001, *B* = 0.22; appraisal, *F*(1,476.6) = 44.88, *p* < 0.001, *B* = 0.44; and valence, *F*(1,461.1) = 15.89, *p* < 0.001, *B* = 0.40. Viewers who felt more moved by the ad content, felt greater physical sensation, appraised the content as more communally sharing, and perceived it as more positive showed more post-ad motivation to support the candidate that commissioned the seen ad.

Additionally, kama muta appraisal and valence were such strong predictors that the interaction effect between viewers’ party preference and the video’s party affiliation became non-significant, *F*(1,447.0) = 2.56, *p* = 0.110 and *F*(1,447.3) = 0.02, *p* = 0.889, respectively. For kama muta feeling and sensation the interaction of party preference and video affiliation remained significant, *F*(1,431.0) = 110.16, *p* < 0.001, *B* = 3.85 and *F*(1,444.6) = 211.24, *p* < 0.001, *B* = 4.11, respectively.

Lastly, the mixed model analyses showed significance for the three-way interactions between the participant’s party preference, the video’s party affiliation and all four emotion components of kama muta, feeling, *F*(1,464.1) = 4.04, *p* = 0.045, *B* = −0.18, sensation, *F*(1,482.9) = 15.37, *p* < 0.001, *B* = −0.30, appraisal, *F*(1,453.1) = 10.44, *p* = 0.001, *p* = 0.045, *B* = 0.46, and valence, *F*(1,446.0) = 13.23, *p* < 0.001, *B* = 0.63, respectively. That means, if a participant’s party preference matched the ad’s party affiliation, the effect of this interaction on the respondent’s motivation to support the candidate was further enhanced by the viewer feeling especially moved by the ad content, especially strong physical sensation, appraising the content as especially communally sharing and perceiving it as especially positive. These interactions were not predicted and contradict earlier findings by Seibt et al. (2019).

### Anger

The analyses were carried out in SPSS with focus on the same two factors as for kama muta analyses: (1) party preference of the participant (Republican vs. Democratic), and (2) the party affiliation of the ad (Republican vs. Democratic). One new factor we included in the analyses for anger, due to not meeting a pre-registered aspect of data quality as explicated above, was the party affiliation participants had guessed for the ad (instead of the video’s actual party affiliation). Identical to kama muta analyses, the dependent variables we were interested in were the strength of emotion evocation (Hypothesis 3) and the motivation to support the ad-commissioning candidate (hypothesis 4).

#### Manipulation Check

First of all, the anger-ads, as expected, upset viewers more than the kama muta-ads did; for anger toward commissioner, *F*(1,1166) = 250.39, *p* < 0.001, $\eta_{\text{p}}^{2}$ = 0.18, and anger against protagonist, *F*(1,1166) = 8.26, *p* = 0.004, $\eta_{\text{p}}^{2}$ = 0.07, appraisal, *F*(1,1166) = 930.49, *p* < 0.001, $\eta_{\text{p}}^{2}$ = 0.44, and negative valence, *F*(1,1166) = 2486.99, *p* < 0.001, $\eta_{\text{p}}^{2}$ = 0.68.

##### Hypothesis 3

###### Analyses With Video’s Guessed Party Affiliation

**FIGURE 2:**
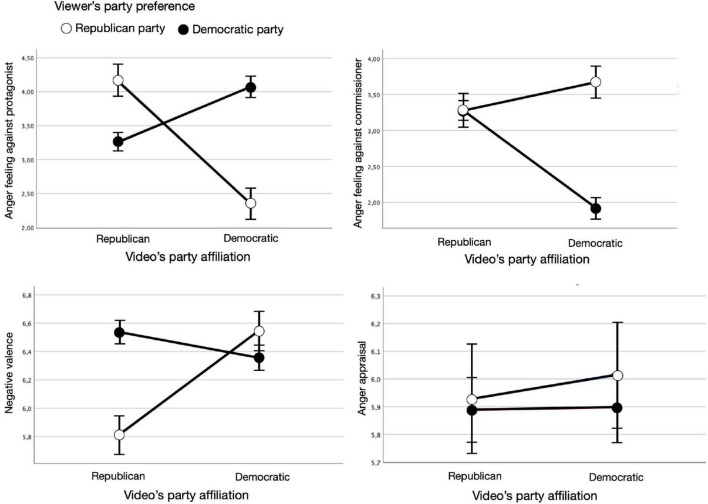
Interaction effects of video’s guessed party affiliation and viewer’s party preference on the four components of anger: feeling against protagonist, feeling against commissioner, negative valence, and appraisal.

Here we used the same mixed model approach as for being moved but using the party affiliation participants guessed for an ad instead of the ad’s actual affiliation. As shown in Figure 2, the four independent analyses showed that the interaction of viewers’ party preference and the ad’s guessed party affiliation had the expected significant effect on three of four emotion components of anger, that is, feeling against protagonist, *F*(1,454.2) = 29.00, *p* < 0.001, *B* = 1.50, feeling against commissioner, *F*(1,450.6) = 28.03, *p* < 0.001, *B* = −2.18, and negative valence, *F*(1,414.8) = 9.75, *p* = 0.002, *B* = −1.27, respectively. Concluding, viewers felt more upset by the featured candidate, less upset by the commissioning candidate, and perceived the ad content as more negative when their party preference was the same as the video’s guessed party affiliation (vs. not the same). The interaction effect, however, was not significant for appraisal, *F* < 1, *p* = 0.426. The video party and the viewer’s party preference only in few instances had a significant main effect on the evocation of all four components.

###### Analyses With Video’s Actual Party Affiliation

**FIGURE 3:**
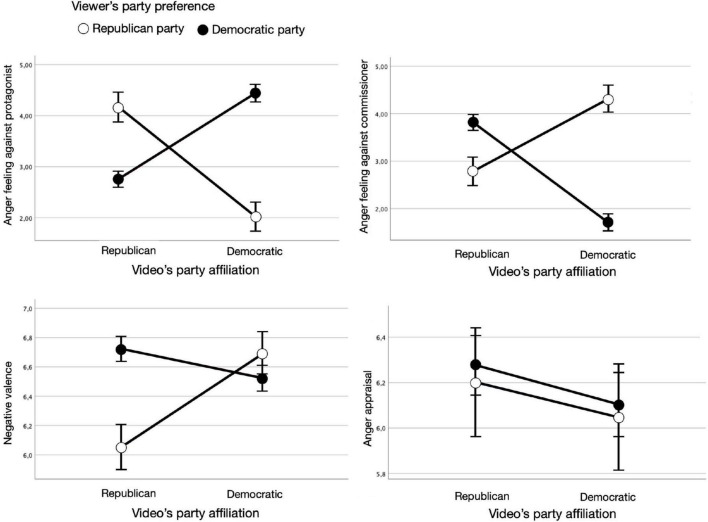
Interaction effects of guessed party affiliation and viewer’s party preference on the four components of anger: feeling against protagonist, feeling against commissioner, negative valence, and appraisal.

Again, we used the same mixed model approach as for the kama muta analyses. For these analyses all cases were excluded in which the party affiliation of a shown ad was indicated incorrectly by a viewer. As shown in Figure 3, the four independent analyses showed that the interaction of viewers’ party preference and the ad’s actual party affiliation had the expected significant effect on three of four emotion components of anger, that is, feeling against protagonist, *F*(1,264.1) = 87.72, *p* < 0.001, *B* = 4.30, feeling against commissioner, *F*(1,253.2) = 106.83, *p* < 0.001, *B* = −4.94, and valence, *F*(1,208.0) = 11.78, *p* = 0.001, *B* = −0.79, respectively. Viewers felt more upset by the featured candidate, less upset by the commissioning candidate, and perceived the ad content as more negative when their party preference was the same as the video’s party affiliation (vs. not the same). The interaction effect, however, was again not significant for appraisal, *F* < 1, *p* = 0.347. The video party had no significant main effect on the evocation of any of the four components and the viewer’s party preference only had a significant main effect on the evocation of the anger feeling against the protagonist of an ad.

##### Hypothesis 4

We used a mixed model with the dependent variable being the motivation to support the candidate commissioning the ad and the independent variables being the video’s guessed or actual party affiliation (Republican vs. Democratic), the participant’s party preference (Republican vs. Democratic), and video ID (1–8). Additionally, the four emotion components of anger (i.e., feeling against protagonist, feeling against commissioner, appraisal, and valence) were used as predictors of viewers’ motivation to support a candidate.

###### Analyses With Video’s Guessed Party Affiliation

Without including the four emotion components explicitly as predictors, the interaction effect of the participant’s party preference and the ad’s guessed party affiliation on the motivation to support a candidate was significant, *F*(1,471.7) = 39.68, *p* < 0.001, *B* = 1.62. That means, if the participant’s party preference and the video’s guessed party affiliation were the same, the motivation to support the candidate that commissioned the ad was enhanced. The main effects of the video’s guessed party affiliation and the viewer’s party preference on the motivation to support showed as non-significant, *F* < 1, *p* = 0.511; and *F* < 1, *p* = 0.482. Specifically, ads which were guessed to be Democratic were not more motivating in general than ones that were guessed to be Republican and vice versa.

Importantly, three out of four emotion components of anger were significant predictors of the motivation outcome of participants: feeling against protagonist, *F*(1,456.4) = 107.96, *p* < 0.001, *B* = 0.45, feeling against commissioner, *F*(1,475.2) = 9.41, *p* = 0.002, *B* = −0.31, and valence, *F*(1,461.1) = 10.10, *p* = 0.002, *B* = 0.02. In short, viewers that felt more upset by the candidate featured in the ad, felt less upset by the ad-commissioning candidate, and perceived the ad as more negative showed enhanced post-ad motivation to support the candidate that commissioned the seen ad. Anger appraisal did not show a significant prediction of a participant’s motivation to support, *F* < 1, *p* = 0.377.

Additionally, felt anger toward the candidate that was featured in the ad (feeling against protagonist) was such a strong predictor that the interaction effect between viewers’ party preference and the video’s guessed party affiliation became non-significant in the process, *F* < 1, *p* = 0.736. In other words, anger completely mediated the effect that a match between the ad’s source and one’s own prior preference had on motivation. Further, anger against the ad-featuring candidate came out as the superior predictor in comparison to anger against the ad-commissioning candidate. Including anger feeling against protagonist and feeling against commissioner in the same analysis lead to a non-significant effect of feeling against commissioner, *F*(1,445.8) = 1.48, *p* = 0.224, and a non-significant interaction of viewers’ party preference and the video’s guessed party affiliation, *F* < 1, *p* = 0.428. Feeling against protagonist held up as a significant predictor, *F*(1,460.4) = 18.42, *p* < 0.001, *B* = 0.45. That means to a viewer’s motivation to support the ad-commissioning candidate it matters how upset a participant feels by the candidate featured in the ad. The side effect of participants getting angry because they don’t agree (with the ad-commissioning candidate or party) doesn’t seem to matter in this case.

The mixed model analyses showed significance for the three-way interactions between the participant’s party preference, the video’s guessed party affiliation and two of four emotion components of anger, that is, appraisal, *F*(1,426.3) = 6.12, *p* = 0.014, *B* = 0.42, and negative valence, *F*(1,482.3) = 9.36, *p* = 0.002, *B* = 0.89, respectively [for anger against protagonist: *F*(1,478.9) = 3.12, *p* = 0.078; and against commissioner: *F* < 1, *p* = 0.861]. That means, if a participant’s party preference matched the ad’s party affiliation, the effect of this interaction on the respondent’s motivation to support the candidate was further enhanced by appraising the ad-content as especially critical and perceiving it as especially negative.

###### Analyses With Video’s Actual Party Affiliation

Without including the four emotion components explicitly as predictors, the interaction effect of the participant’s party preference and the ad’s actual party affiliation on the motivation to support a candidate was significant, *F*(1,280.8) = 80.01, *p* < 0.001, *B* = 2.56. That means, if the participant’s party preference and the video’s actual party affiliation matched, the motivation to support the candidate that commissioned the ad was enhanced. Further, the video’s actual party affiliation had a main effect on the motivation to support, *F*(1,280.8) = 7.57, *p* = 0.006, *B* = −1.67. Specifically, Democratic ads were more motivating in general (*M* = 2.77) than Republican ones (*M* = 1.80). The main effect of viewer’s party preference was non-significant, *F*(1,111.7) = 1.05, *p* = 0.308.

Importantly, three out of four emotion components of anger were significant predictors of the motivation outcome: feeling against protagonist, *F*(1,313.5) = 73.60, *p* < 0.001, *B* = 0.49; feeling against commissioner, *F*(1,300.9) = 4.12, *p* = 0.043, *B* = −0.31; and valence, *F*(1,253.9) = 6.13, *p* = 0.014, *B* = 0.17. Again, as for ads’ guessed party affiliation, viewers that felt more upset by the candidate featured in the ad, felt less upset by the ad-commissioning candidate, and perceived the ad as more negative showed enhanced post-ad motivation to support the candidate that commissioned the seen ad. Anger appraisal did not show a significant prediction of the participant’s motivation to support, *F*(1,173.6) = 1.21, *p* = 0.273.

Additionally, and similarly to analyses with a video’s guessed party affiliation, anger feeling against protagonist was such a strong predictor that the interaction effect between viewers’ party preference and the video’s party affiliation showed as non-significant in the process, *F* < 1, *p* = 0.858. Further, feeling against protagonist came out as the superior predictor in comparison to feeling against commissioner. Including anger feeling against protagonist and feeling against commissioner in the same analysis lead to a non-significant effect of feeling against commissioner, *F*(1,260.8) = 2.23, *p* = 0.136, and a non-significant interaction of viewers’ party preference and the video’s actual party affiliation, *F*(1,431.6) = 2.53, *p* = 0.113. Feeling against protagonist held up as a significant predictor, *F*(1,276.1) = 16.17, *p* < 0.001, *B* = 0.51. That means, identical to the findings for ads’ guessed party affiliation, to predict a viewer’s motivation to support the ad-commissioning candidate it is irrelevant to know how upset this participant is with the candidate that commissioned the ad, if one knows how upset a participant feels with the candidate featured in the ad.

Lastly, the mixed model analyses showed significance for the three-way interactions between the participant’s party preference, the video’s actual party affiliation and three of four emotion components of anger, feeling against protagonist, *F*(1,305.3) = 6.48, *p* = 0.011, *B* = 0.40, appraisal, *F*(1,279.3) = 4.932, *p* = 0.03, *B* = 0.42, and negative valence, *F*(1,304.7) = 7.85, *p* = 0.005, *B* = 0.89, respectively (against commissioner: *F* < 1, *p* = 0.933). As in the analyses with an ad’s guessed party affiliation, that means, if a participant’s party preference matched the ad’s party affiliation, the effect of this interaction on the respondent’s motivation to support the candidate was further enhanced by feeling more anger toward the ad-featured candidate, appraising the ad-content as especially critical and perceiving it as especially negative.

## Discussion

The present study was conducted before the United States midterm elections of 2018. Participants were shown eight ads from real political campaigns of the midterm elections. We first tested if the ads evoked stronger emotions of *being moved* and *anger* in a viewer who watched ads that support the party she or he identifies with most. Second, we tested if these evoked emotions significantly increased the motivation to support a candidate.

The emotion *being* moved, that is all its four components, were significantly affected by the interaction effect of the party resembled by the video and the participant’s party preference. If one’s party affiliation matched the source of the video, participants experienced more kama muta. Further, for all emotion components (i.e., feeling, sensation, appraisal, and valence) their evocation significantly predicted the participant’s motivation to support the focused candidate and party.

For *anger* we found the same effect pattern, except for a non-significant emotional effect on and motivational effect of anger’s constructed appraisal measure. The other emotion components (i.e., feelings and valence) were significantly evoked by the interaction effect of the party which commissioned the ad and the participant’s party preference and these emotion evocations significantly predicted the motivation of participants to support the candidate and party that commissioned the ad.

For both emotions we find a three-way interaction effect of the video’s party, the participant’s party preference and the emotion evocation on the viewer’s support motivation. That is, the present paper points at two moderation effects of video party and party preference. First, people are more moved and angered by videos of the party they identify with. Second, to the extent that one experiences kama muta, that experience motivates more strongly to support the party if it is one’s own party.

What can we learn from the results? First, people get especially moved or angered by political ads that are commissioned by the party they identify with more. Second, these two evoked emotions, more specifically their different components, influence a participant’s motivation to support the commissioning party and its candidate, in direct (i.e., voting for the candidate) as well as ideational (e.g., recommending the party to others) and financial terms (e.g., donating money to the party). The evoked emotions motivate a viewer more if they are evoked by a video from the party the viewer identifies with.

The present results are relevant in five ways. First, by replicating the results of Seibt et al. (2019) we support their reliability, solidifying our understanding of the theoretical concept of kama muta and its effects in political advertisement. Second, in our study we focused on ads that were much shorter than in previous research and chose the United States midterm instead of presidential elections that have been in prominent focus so far (see, e.g., Devlin, 1995; Benoit, 1999; Goldstein and Wilner, 2012). This focus expands the generalizability of the supported emotion effects, enhancing the theory’s external validity. Third, the present study makes the point that an ad length substantially shorter than the common presidential campaign ads is already sufficient for a significant evocation of being moved or anger in viewers. On top of that, these political spots are able to facilitate a relevant influence on participants’ motivation for different ways of political support. Finding these emotional effects advances existing insights on the impact of short attack-ads on cognitive responses (e.g., Kern and Just, 1995; Bradley et al., 2007). Note, however, that the comparison of effects in the present study with the effects Seibt et al. (2019) report is confounded by the ad-content, which was different. A direct comparison of these two is, hence, not informative.

Fourth, we also find a significant three-way interaction of the video party, viewer’s party preference and the emotion evocation in their prediction of the participant’s support motivation. This means that evoked kama muta only translated into support if the source of the emotion was an ad by one’s already initially preferred party. This interaction was not observed by Seibt et al. (2019). There, the conclusion was that kama muta is able to overcome group boundaries [see also recent work by Blomster Lyshol et al. (in press)]. It is, however, in line with arguments that self-relevance is of importance (Mujani and Liddle, 2010; Chou and Lien, 2011). Methodological reasons might be that Seibt et al. (2019) used political ads that were longer than the videos used in the present study and that were material for the presidential election rather than the more partisan midterm election. Both factors might have enhanced the videos’ emotional influence on viewers leaving less room for a three-way interaction effect to show as significant.

Lastly, while anger has long been recognized as an important emotion in the political context (Marcus, 2003; Brader, 2005), the present findings regarding anger effects of political ads are the first of their kind we are aware of. We enable the first parallel comparison of a positive social-relational emotion and anger in their evocation by political advertisement and their subsequent effects on the motivation for political support. By this comparison, we show that anger, like kama muta, can be represented and assessed from different emotion components and empirically support the notion in literature that anger is a motivator for political support.

### Limitations and Future Research

The appraisal of anger, as the only component in the study’s array of emotion components, was neither significantly affected by political ads nor did it have an effect on people’s motivation to support a political campaign. A possible explanation might be that the wording of the appraisal item did not encode the correct kind of appraisal for anger. The item we chose to measure the appraisal of critique and rejection has measured exactly that. However, to correctly display an anger appraisal we argue that the item ought to not just capture critique and rejection but *unjust* critique and rejection instead. The appraisal item ought not to ask if there is critique on and rejection of a candidate, but if the critique or the rejection are unjust. To combat the measurement issue of the anger appraisal we propose to extend the measurement of appraisal to more than one item and to include items that do not encode the appraisal of critique and rejection (i.e., “There were people in the ad who were criticized and rejected.”) but rather the appraisal of unjust critique and rejection (i.e., “There were people in the ad who were *unjustly* criticized and rejected.”). Alternatively, or in addition, the appraisal item could tap into goal blocking and goal frustration in general, in line with prevalent notions of the anger appraisal (Kuppens et al., 2003, 2007).

While we found a significant interaction effect between the ad’s party affiliation and the viewer’s party preference on a viewer’s feeling of being moved, on a more detailed level only Democratic sympathizers show a significant difference in being moved and angered by watching Democratic vs. Republican ads. Republican sympathizers are descriptively, but not significantly, more moved and angered by Republican than Democratic ads. This might have two reasons. First, the sample size of Republican sympathizers (*N* = 41) might have been too low to provide a reasonable power for an interaction analysis to hold significant results. However, the descriptives also do not point to the conclusion that Republican sympathizers, like Democratic ones, show more emotions when watching the ad their preferred party commissioned. Second and more aligning with descriptives, there could be a difference between Republican and Democratic sympathizers or the ads themselves in being moved by political ads. However, answering such a question with satisfying certainty would need future research with higher statistical power to rule out the former shortcoming of the present study, and more control over the stimulus materials.

On the more detailed level of party sympathy we, further, find that while Republican sympathizers are more angered by attack ads against the Democratic than against the Republican party, this anger does not motivate them to support the Republican party instead of the Democratic Party. Again, two explanations can make sense of this phenomenon. The statistical power was too low^5^ or there might be an actual difference in anger-motivated political support between Republican and Democratic sympathizers. Yet again, further research has to test both assumptions.

The present study did not include sensation measures for anger. Such measures should be implemented in future research to facilitate a more extensive view on the emotion concept of anger and enhance the resolution of anger’s effect pattern in political advertisement.

We are aware of the weaknesses of motivation measures and the assessment of intentions (see for other cases Chou and Lien, 2010, 2011). Their validity as replacement of actual behavior is of course limited. We expect it to be difficult to completely break away from an artificial design and measure real voting behavior of participants in the election; first and foremost, this will be difficult because of confidentiality issues connected to recording people’s votes. However, looking at the different ways political support was measured in the present study, other measures of voting-related behavior are certainly possible to implement. For example, future studies might measure how many other people a participant contacts to praise a candidate or how much a candidate donates to the ad-commissioning candidate after having seen a respective political spot (see for similar application, Erlandsson et al., 2018). Behavioral measures are central to understand the range of effects emotions have on people’s voting behavior because they analyze the impactful step after an intention has formed. Note also that within-designs and measures on interval scales provide more statistical power.

Moreover, recent research has shown that the motivational effect of a single ad might be small on average (Coppock et al., 2020). We have presented participants with a conglomerate of political videos per emotion and political party. Future research may want to examine potential cascade effects of subsequently watching several political ads.

Lastly, a growing literature on examining the cognitive antecedents of evaluating leading vs. trailing political candidates (e.g., Alves and Mata, 2019; Grüning et al., 2021) raises the question of how this feature of an election may affect emotion evocation and motivational effects. Potentially, the evocation of being moved is especially pronounced for a political party that is behind in votes because this situation accentuates the problem that has to be overcome by working together. Future research might explore this and other elements that characterize political elections beyond the identification with a political side.

### Practical Implications

The interaction effect of the participant’s and the video’s party affiliation held up even when participants guessed an ad’s affiliation. That is, for a relevant emotional influence it did not matter if the party affiliation that matched the viewer’s preference was an actual or a constructed one. This has several practical implications for advertisement for political campaigns. For instance, to just name one, campaign ads would do well to unambiguously communicate their political affiliation to prevent unwillingly feeding the support of political competitors.

On another practical note, in regard to policy making, the question arises how powerful and, consequently, how dangerous emotionalized political ads can be for the voter. If behavioral studies support the present findings of motivational effects on voter support, underpinning the manipulative potential of emotionalized political ads, multiple practical questions would urgently call for answers. For instance, should a political ad’s emotionalizing potential be explicitly evaluated and communicated? We can not give answers from our current results, but any democratic process should be aware of the power of both heartwarming and attack ads to persuade through emotions.

Finally, the moderation of an ad’s impact on emotions, and the subsequent moderation of that emotion’s impact on motivation by the source of the video supports that ads in a modern political landscape actually have little effect in terms of winning over voters from the other side (but see, Phillips et al., 2008). Instead, they have impact on constituents who already identify to support through donations, and to turn out to the ballot box.

## Conclusion

Our findings provide a glimpse into how the emotional side of political advertisement works. The present paper finds a double moderation of a video’s party affiliation and the viewer’s party preference. If the party that commissions the political ad fits the viewer’s party preference the video evokes more kama muta and anger, depending on the video, in the viewer than if video party and party preference do not fit. Importantly, this same fit then also plays a role for the effect an evoked emotion has on the motivation of the viewer to support a candidate or party directly (i.e., voting for the candidate), ideationally (e.g., recommending the party to others) and financially (e.g., donating money to the party). That is, the fit between the video’s party affiliation and the viewer’s party preference matters for the emotional as well as, subsequently, the motivational influence of a political ad. In conclusion, the present findings show that political ads mainly influence voting turn out and alternative political support. However, they are less effective in changing people’s minds and overtaking voters from the other political side.

## Data Availability Statement

The dataset presented in this study can be found in the following online repository: https://osf.io/v3ecg/.

## Ethics Statement

The study involved human participants and was, therefore, reviewed and approved by the internal review board at the Department of Psychology, University of Oslo. The participants provided their written informed consent to participate in this study.

## Author Contributions

DG created the materials, ran the study, analyzed the data, and wrote the first draft. DG and TS revised materials, checked analyses, and revised the manuscript. Both authors jointly planned the study and the analyses.

## Conflict of Interest

The authors declare that the research was conducted in the absence of any commercial or financial relationships that could be construed as a potential conflict of interest.

## Publisher’s Note

All claims expressed in this article are solely those of the authors and do not necessarily represent those of their affiliated organizations, or those of the publisher, the editors and the reviewers. Any product that may be evaluated in this article, or claim that may be made by its manufacturer, is not guaranteed or endorsed by the publisher.

## Footnotes

1
https://osf.io/v3ecg/

2 We refer to the definition by Hareli and Parkinson (2008) that social appraisals have to be involved as causes or constituents for an emotion to be considered as social.

3
https://aspredicted.org/25598.pdf

4 We included this variable to cover the case that people get angry at a political ad they don’t agree with.

5 Note that in this case the descriptives, indeed, point in the direction of the hypothesized effect.
